# From Waste to Value: Sustainable Development of Functional Pasta Enriched with Pumpkin Peel Powder and Its Nutritional Assessment

**DOI:** 10.3390/molecules31152637

**Published:** 2026-07-29

**Authors:** Małgorzata Stryjecka, Adam Cudowski, Anna Kiełtyka-Dadasiewicz

**Affiliations:** 1 Institute of Human Nutrition and Agriculture, The University College of Applied Sciences in Chełm, 22-100 Chełm, Poland; mstryjecka@panschelm.edu.pl; 2 Department of Water Ecology, Faculty of Biology, University of Bialystok, Ciolkowskiego 1J, 15-245 Bialystok, Poland; cudad@uwb.edu.pl; 3 Department of Plant Production Technology and Commodity Sciences, University of Life Sciences, 20-950 Lublin, Poland

**Keywords:** pumpkin peels, *Cucurbita maxima*, functional food, pasta, dietary fiber, phenolic compounds, antioxidant activity, by-product valorization

## Abstract

Given the increasing need to reduce food losses and implement circular economy strategies, plant-based processing byproducts represent a promising source of functional ingredients. The aim of this study was to comprehensively assess the potential of pumpkin peels as a raw material for enriching cereal products, as a value-added ingredient for wheat pasta, and to analyze their nutritional and technological properties. Pumpkin peels were dried, ground, and thoroughly analyzed for chemical composition, bioactive compound content, and functional properties. Model pastas were developed with a 5–15% addition of pumpkin peel powder and assessed for their chemical composition, cooking quality, rheological behavior, antioxidant properties, starch digestibility, texture, oxidative stability, and in vitro prebiotic potential, as well as their health-promoting potential. The results demonstrated that pumpkin peel powder was a rich source of dietary fiber, polyphenols, and carotenoids, which translates into high antioxidant activity. Enriching the pasta with this raw material significantly increased the content of bioactive components and fiber, while having a moderate impact on technological properties. The most beneficial effects were achieved with a 10–15% addition, although higher levels may affect texture and cooking parameters. Pumpkin peels can therefore be a valuable ingredient in functional foods, supporting waste valorization and circular bioeconomy strategies.

## 1. Introduction

Growing concerns over climate change, rapid population growth, and the depletion of natural resources, have intensified the need to transform current food systems toward more sustainable production and consumption patterns. Among the major challenges facing the agri-food sector is the reduction in food loss and waste, which is estimated to account for nearly one-third of all food produced worldwide [1,2,3].

In response to this problem, strategies based on the principles of the circular economy are being developed, aiming to maximize the utilization of raw materials and the reuse of by-products generated throughout the food processing chain. Particular attention is currently being paid to the potential of plant biomass, which has until now been treated as waste or as a raw material of low utility value [4,5,6]. By-products such as peels, seeds, and pomace constitute valuable sources of bioactive components that can be reused in the food, pharmaceutical, and cosmetic industries [7,8,9,10].

In this context, identifying new functional raw materials and developing effective methods for their valorization are crucial. Pumpkin (*Cucurbita* spp.) is a plant widely distributed worldwide and valued for both its nutritional and technological benefits. Pumpkin fruits are rich in vitamins, minerals, and bioactive compounds, including carotenoids and polyphenols [11,12]. They are used in the production of processed foods such as purees, juices, bread, and pasta. However, the technological processes associated with pumpkin processing generate significant amounts of waste, a substantial proportion of which consists of peels. Despite their availability and potential value, pumpkin peels remain largely unused or are utilized only to a limited extent, for example, as animal feed or compost.

In recent years, there has been growing scientific interest in the chemical composition of pumpkin peels and their potential application in functional foods. Research indicates that these peels are a rich source of dietary fiber, both soluble and insoluble, which plays a significant role in regulating digestive function and preventing lifestyle-related diseases [13,14,15,16,17]. Furthermore, they contain significant amounts of phenolic compounds that exhibit strong antioxidant properties and may contribute to reducing oxidative stress in the body [18]. Another important characteristic of pumpkin peels is the presence of carotenoids, including β-carotene, which acts as provitamin A and exerts protective effects on body cells. These compounds are important not only because of their nutritional value but also as natural colorants that can be used in the production of foods with enhanced sensory appeal. Additionally, pumpkin peels contain minerals such as potassium, magnesium, and calcium, which play key roles in the proper functioning of the human body.

Besides their nutritional value, pumpkin peels also exhibit a number of functional properties that are important from a technological perspective. Their high fiber content enhances their water- and fat-binding capacities, which can improve the texture and stability of food products. Incorporating these raw materials into food formulations may also contribute to extending product shelf life by reducing lipid oxidation [19]. Therefore, pumpkin peels can serve as an attractive functional ingredient in the production of high-value foods.

In the context of sustainable development, the concept of valorization is particularly important, referring to the creation of added value from by-products through their processing and use in innovative applications [20]. In the case of pumpkin peels, valorization strategies may include their incorporation into cereal-based products (e.g., bread and pasta), their use as a component of dietary supplements, and their application as a source of extracts rich in bioactive compounds [9]. This approach not only reduces waste but also increases the economic efficiency of production processes.

Despite growing interest in this area, significant research gaps remain regarding the comprehensive assessment of the potential of pumpkin peels. Previous studies have often focused on selected aspects, such as chemical composition or antioxidant properties, while overlooking their broader application in food systems [3]. There is also a lack of standardized analytical methods and application-oriented studies that would facilitate the implementation of these raw materials on an industrial scale. Therefore, the aim of this study is to comprehensively analyze the chemical composition and functional properties of pumpkin peels powder (PPP), and to evaluate its suitability as a functional ingredient in wheat pasta. Particular attention was paid to determining the optimal level of PPP incorporation that provides the greatest nutritional benefits while maintaining acceptable technological and sensory-related properties.

To the best of our knowledge, this is the first such comprehensive study investigating the effects of pumpkin peel powder on pasta properties, including, in addition to its composition and nutritional and antioxidant properties, the following: cooking and technological properties of pasta, rheological properties of pasta dough, starch digestibility and predicted glycemic index, in vitro bioaccessibility of phenolic compounds, texture profile analysis (TPA) of cooked pasta, oxidative stability during storage of pasta enriched with PPP, and in vitro prebiotic potential of enriched pasta.

## 2. Results and Discussion

### 2.1. Chemical Composition of Pumpkin Peels Powder (PPP) as a Determinant of Functional Properties

Analysis of the chemical composition of the powder obtained from pumpkin peels showed that the tested material was characterized by a very high dry matter content of 92.4% (Table 1). This indicates a low moisture content, which is beneficial for product shelf life and storage stability. The protein content of the tested powder reached 8.7%, while the fat content was 3.1%, indicating a moderate nutritional value in terms of macronutrients. The ash content (6.5%), reflecting the presence of minerals, also constituted a significant component of the chemical composition. The most distinctive feature of the analysed raw material was its very high dietary fiber content (42.8%). Furthermore, a considerable amount of bioactive compounds was detected, including polyphenols (18.6 mg GAE/g dry matter) and carotenoids (21.3 mg/100 g dry matter), which are known for their antioxidant properties.

The obtained results indicate that pumpkin peel powder can be a valuable functional raw material. The high dry matter content is consistent with literature data on dried plant products and confirms the effectiveness of the drying process [3,18]. The protein content of 8.7% can be considered moderate; however, compared with other plant by-products, it is relatively favorable. The low fat content confirms that this product may be used in the formulation of reduced-calorie foods [21].

The very high dietary fiber content is particularly noteworthy. A value exceeding 40% suggests that pumpkin peel powder can be effectively used as an ingredient to enrich food products with dietary fiber, which is important in the prevention of digestive and metabolic diseases [22]. The ash content indicates the presence of minerals, which further enhances the nutritional value of the tested material. The presence of polyphenols and carotenoids confirms the antioxidant potential of the powder. These compounds can neutralize free radicals, thereby helping to reduce oxidative stress in the body [23]. It is worth noting that the polyphenol content is comparable to that of other plant foods considered rich in antioxidants. The carotenoid content, in turn, indicates that pumpkin peels, often treated as waste, may represent a valuable source of natural pigments and health-promoting compounds.

### 2.2. Functional Properties of Pumpkin Peel Powder (PPP)

The functional properties of pumpkin peel powder (PPP) indicate its high capacity to interact with water and lipids, as well as its considerable technological potential in food products (Table 2). The water-holding capacity (WHC) was 4.82 g water/g sample, indicating the material’s high hygroscopicity. The oil-holding capacity (OHC) reached 2.36 g oil/g sample, indicating a moderate ability to retain lipids. The swelling index was high (5.9 mL/g), while the swelling capacity reached 41.2%. Emulsion stability was 38.5%, suggesting a limited but noticeable ability of PPP to stabilize emulsion systems.

The obtained results indicate that pumpkin peel powder possesses beneficial functional properties that can be utilized in food technology, particularly in cereal-based and functional food products. The high WHC value is directly related to the presence of dietary fiber, particularly the insoluble fraction, which contains numerous hydrophilic groups capable of binding water molecules [14,22,24]. Water-holding capacity has important technological implications, as it influences process efficiency, texture, and product freshness. In the case of pasta, it may contribute to improved moisture retention and reduced cooking losses. Similar properties have been reported for plant-based fiber additives [18,25,26,27].

The OHC value indicates that PPP can interact with the lipid fraction, which is important for the flavor and textural stability of food products. Oil-holding capacity is often attributed to the fibrous structure of plant materials and the presence of components such as lignin and hemicelluloses [28]. Although the obtained value is not particularly high, it is sufficient to influence sensory properties, such as juiciness and flavor intensity.

Swelling capacity is another important parameter determining the functionality of PPP. A high value of this parameter indicates the material’s ability to increase in volume in the presence of water, which is of both physiological and technological importance. From a nutritional perspective, it may increase satiety, whereas in food technology, it affects product structure and viscosity [22,24].

An emulsion stability of 38.5% suggests a moderate ability of PPP to stabilize oil–water systems. This property may be attributed to the presence of polysaccharides and residual proteins, which can act as natural emulsifiers. However, compared with traditional functional additives (e.g., plant proteins or hydrocolloids), this ability is limited, indicating that PPP may play a supporting rather than a dominant role in emulsion stabilization.

From a technological perspective, the combination of high water-holding capacity and moderate oil-holding capacity indicates that PPP can be effectively used as an ingredient to improve the texture and stability of food products. In the case of pasta, this may lead to changes in dough structure, increased viscosity, and modifications of cooking parameters [29,30]. Furthermore, the functional properties of PPP are consistent with current trends in the utilization of food processing by-products in the food industry. Fiber-rich materials, such as vegetable peels, represent a valuable source of functional ingredients and may serve as natural alternatives to synthetic additives [9].

Pumpkin peel powder is characterized by high water-holding capacity and good swelling capacity, making it a valuable functional ingredient. Its moderate emulsifying properties and oil-holding capacity further enhance its application potential. PPP can be successfully used in food products as a natural ingredient that improves texture, stability, and nutritional value.

### 2.3. Change in the Composition of Pasta After Enriching It with Pumpkin Peel Powder (PPP)

Enriching pasta with pumpkin peel powder (PPP) significantly affected its basic composition (Table 3). The dry matter content showed a slight but systematic decrease with increasing PPP addition (from 88.28% in the control sample to 87.72% in the sample containing 15% PPP). This change was small and within the typical range for pasta products, indicating that PPP addition does not significantly affect the product’s moisture stability. The protein content decreased proportionally with the level of flour substitution (from 12.34 to 11.21 g/100 g DM). This is due to the fact that pumpkin peels contain less protein than wheat flour, resulting in a protein dilution effect in the final product. At the same time, an increase in fat content was observed (from 1.48 to 1.74 g/100 g DM), which can be attributed to the presence of lipids in the plant material, particularly in the fractions associated with the peel tissues. A significant increase was also observed in ash content (from 0.95 to 1.32 g/100 g DM), indicating an increased higher mineral content. However, the most pronounced changes were observed in the dietary fiber content. Total dietary fiber content almost tripled (from 3.33 to 8.89 g/100 g DM), with insoluble fiber being the dominant fraction. Its content increased from 2.43 to 6.83 g/100 g, while soluble fiber increased moderately (from 0.90 to 2.06 g/100 g).

Pumpkin peel powder is an effective functional ingredient that improves the nutritional value of pasta, particularly in terms of dietary fiber and mineral content. The increase in ash content is consistent with previous reports indicating that vegetable by-products are a rich source of micro- and macronutrients [18]. A decrease in protein content is a typical effect observed when wheat flour is partially replaced with plant-based ingredients containing lower protein levels. Similar trends have been reported by other authors investigating the enrichment of pasta with fiber-rich raw materials, such as bran or vegetable waste [31]. From a technological perspective, a reduction in protein content may affect the rheological properties of the dough and the quality of the final product, particularly its structure and elasticity.

The most significant finding is the substantial increase in dietary fiber content. Pumpkin peels are particularly rich in insoluble fiber, which explains the observed predominance of this fraction. According to the literature, insoluble fiber plays a key role in regulating intestinal peristalsis and preventing gastrointestinal diseases [22]. In turn, the presence of soluble fiber, although in smaller amounts, may contribute to lowering cholesterol levels and improving the glycemic response. The increase in fat content, although statistically significant, remained low in absolute terms and did not adversely affect the nutritional profile of the product. However, it may be important for sensory properties, such as taste and mouthfeel, as suggested by previous studies on the incorporation of plant materials into cereal products [28,32,33,34,35].

The slight decrease in dry matter content can be explained by the higher water-binding capacity of the dietary fiber present in PPP. The high hygroscopicity of dietary fiber promotes greater water retention within the product structure, which is consistent with the observations reported by other authors investigating additives rich in non-starch polysaccharides [25]. From a nutritional perspective, enriching pasta with 10–15% PPP appears to be particularly beneficial, as it significantly increases the dietary fiber content without drastically reducing the protein content. Such products can be classified as functional foods and are consistent with current trends in food industry waste valorization within the framework of the circular economy concept [9].

The addition of PPP effectively improves the nutritional value of pasta, particularly by increasing its dietary fiber and mineral contents. At the same time, it moderately reduces the protein content, which is a technologically predictable effect. The optimal enrichment level appears to be in the 10–15% range, providing a favorable compromise between nutritional quality and potential changes in technological properties.

Although enrichment levels of 10–15% provided the greatest nutritional improvement, the accompanying reduction in protein content and modification of the gluten matrix indicate that nutritional enhancement inevitably involves technological trade-offs. Therefore, the optimal level of PPP incorporation should be selected according to the intended product characteristics and target consumer group rather than maximizing bioactive compound content alone.

### 2.4. Bioactive Compounds and Antioxidant Activity of Pasta Enriched with PPP

Enriching pasta with pumpkin peel powder (PPP) significantly increased the content of bioactive compounds and the antioxidant activity of the product (Table 4). The total phenolic compound content increased proportionally with the level of PPP addition, from 2.50 mg GAE/g d.m. in the control sample to 4.92 mg GAE/g d.m. in the sample containing 15% PPP, representing an almost six-fold increase. A similar trend was observed for carotenoids. Their content increased more than ten-fold, from 0.47 mg/100 g to 3.59 mg/100 g, with increasing PPP levels. Antioxidant activity, assessed using the DPPH and ABTS assays, also increased with the level of PPP addition. For DPPH, the values increased from 4.87 to 19.94 µmol TE/g, while for ABTS, they increased from 5.40 to 22.73 µmol TE/g. In both assays, a nearly linear relationship was observed between the amount of PPP and the free radical scavenging capacity.

Pumpkin peels are a valuable source of bioactive compounds that can be effectively incorporated into cereal products. The significant increase in total phenolic content is consistent with previous studies demonstrating that vegetable by-products are rich in these compounds, particularly in the outer layers of plant materials [18]. Polyphenols are among the most important natural antioxidants, capable of neutralizing reactive oxygen species and reducing oxidative stress in the body. An increase in their content in pasta enriched with PPP directly translates into enhanced antioxidant activity, as confirmed by the results of the DPPH and ABTS assays. A similar relationship between phenolic compound content and antioxidant activity has also been reported by other authors [22].

A substantial increase in carotenoid content was also observed as a result of PPP enrichment. These compounds, including β-carotene, are characteristic constituents of pumpkin and are responsible for both antioxidant activity and provitamin A function [36]. Such a marked increase in their content indicates that PPP may serve as an effective ingredient for enhancing the health-promoting value of cereal products, particularly in diets low in natural antioxidants [37,38,39,40].

Both the DPPH and ABTS assays demonstrated a significant increase in antioxidant activity with increasing PPP levels. Although the two methods are based on different reaction mechanisms, the obtained results are consistent and indicate the strong free radical scavenging capacity of the tested samples. The literature emphasizes that the use of more than one analytical method increases the reliability of antioxidant potential assessment in food products [41,42].

It is worth emphasizing that the observed increase in antioxidant activity has not only nutritional but also technological implications. Antioxidant compounds can improve the oxidative stability of the product by delaying lipid oxidation and pigment degradation, which may have a beneficial effect on the shelf life of pasta [28,43]. From the perspective of food functionality, the obtained results are consistent with current trends in the development of products with an increased content of natural antioxidants. The use of pumpkin peels as a secondary raw material is also consistent with the principles of the circular economy and food waste reduction [9,44].

The addition of pumpkin peel powder significantly increases the content of bioactive compounds and the antioxidant activity of pasta. The greatest benefits are observed at PPP addition levels of 10–15%, where a high concentration of polyphenols and carotenoids, together with a significant increase in free radical scavenging capacity, is achieved. Such a product can be classified as a functional food with enhanced health-promoting potential.

These findings demonstrate that pumpkin peel powder can effectively increase the antioxidant potential of pasta. Nevertheless, the biological relevance of these improvements should be interpreted with caution because antioxidant activity determined by chemical assays does not necessarily reflect physiological efficacy after human consumption. Therefore, additional in vivo investigations are required to confirm the potential health benefits.

### 2.5. Technological Properties of Pasta Enriched with Pumpkin Peel Powder (PPP)

Analysis of the technological properties of pasta enriched with pumpkin peel powder (PPP) revealed a significant effect of the additive on the cooking parameters (Table 5). The cooking time gradually decreased with increasing PPP levels, from 9.5 min in the control sample to 8.8 min in the sample containing 15% PPP. At the same time, a systematic increase in cooking loss was observed, rising from 5.1% to 7.8%. These values are still within the acceptable range for pasta but indicate a deterioration in the product’s structural integrity. Water absorption capacity increased with PPP addition, from 145% in the control sample to 168% at the highest enrichment level. This indicates an enhanced ability of the pasta to bind water during cooking.

The shortened cooking time of PPP-enriched pasta can be explained by the weakening of the gluten structure resulting from the partial replacement of wheat flour with fiber-rich plant material. The gluten network is responsible for forming a compact structure that limits water penetration; therefore, its disruption accelerates the hydration and starch gelatinization processes [31]. Increased cooking losses are commonly observed in fiber-enriched products. The higher amount of soluble substances released into the cooking water may result from the weakening of the protein–starch matrix and its reduced ability to retain soluble components [28,45,46,47]. Despite the increase in this parameter, the obtained values did not exceed the levels considered critical for pasta quality, suggesting that the product retained acceptable technological properties.

A significant effect of PPP addition was the increased water absorption capacity. This phenomenon is primarily attributed to the high dietary fiber content, which is capable of binding large amounts of water due to the presence of numerous hydrophilic groups [22]. From a technological perspective, increased water absorption may increase the weight of the product after cooking and modify its texture, often resulting in a softer structure.

It is worth emphasizing that the relationship between increased water absorption and higher cooking losses is interrelated. Greater hydration of the pasta structure promotes the diffusion of soluble components into the cooking water, which may explain the simultaneous increase in both parameters. Similar observations have been reported in studies on pasta enriched with high-fiber plant additives [25,48,49]. From the perspective of consumer acceptance, these changes represent a compromise. On the one hand, increased water absorption and shorter cooking times may be perceived as practical advantages; on the other hand, increased cooking losses may negatively affect texture and the clarity of the cooking water.

Considering all the evaluated parameters, a PPP addition level of 5–10% appears to be optimal, as it allows improved functional properties while causing only moderate technological changes. Higher addition levels (15%) lead to more pronounced structural changes, which may require further optimization of the technological process.

From an industrial perspective, these observations indicate that PPP enrichment should be accompanied by optimization of processing conditions, particularly water addition and extrusion parameters. Such technological adjustments may allow higher incorporation levels while minimizing adverse effects on cooking quality.

### 2.6. The Effect of the Additive Pumpkin Peel Powder on the Rheological Properties of Pasta Dough

The rheological properties of the pasta dough changed significantly with increasing levels of pumpkin peel powder (PPP) addition (Table 6). The storage modulus (G′) increased systematically, from 1450 Pa in the control sample to 1890 Pa in the sample containing 15% PPP, indicating an increased contribution of elastic properties to the dough structure. At the same time, an increase in the loss modulus (G″) was observed, with values rising from 420 to 580 Pa. This indicates that the viscous component of the system also increased with PPP addition. Dough viscosity increased proportionally with the level of PPP addition, from 38.5 to 50.3 Pa·s, indicating greater resistance to flow and a more compact structure.

The increase in both the storage modulus (G′) and the loss modulus (G″) indicates that PPP addition leads to an overall strengthening of the dough structure while maintaining its viscoelastic character. The predominance of G′ over G″ in all samples suggests that the doughs retained a predominantly elastic character, which is typical of gluten-containing systems. However, the increase in both parameters also indicates greater stiffness of the system [31,50].

The increase in G′ can be explained by the presence of dietary fiber, which acts as a structural filler, limiting molecular mobility and strengthening the dough network. Dietary fiber, particularly the insoluble fraction, can form physical interactions with the gluten–starch matrix, leading to increased resistance to deformation [22,51]. At the same time, the increases in G″ and viscosity indicate a greater contribution of the viscous phase, which may result from the higher water-binding capacity of PPP. Fiber hydration leads to an increase in the amount of water bound within the system, thereby increasing viscosity and restricting flow [52,53]. From a technological perspective, increased dough viscosity may have both positive and negative consequences. On the one hand, a more viscous dough may improve dough cohesion and facilitate product formation. On the other hand, excessive viscosity may complicate extrusion and processing, particularly at higher PPP addition levels [54]. From a practical perspective, PPP addition levels of up to 10% appear to provide a favorable balance between improved functional properties and adequate dough workability. At a PPP level of 15%, the observed increase in viscosity and rheological moduli may require modifications to technological parameters, such as water content or mixing conditions.

### 2.7. Change in the Color of Pasta Due to the Addition of PPP

The addition of pumpkin peel powder (PPP) to pasta resulted in significant changes in the color parameters measured in the CIE Lab* system (Table 7). The L* parameter (lightness) decreased systematically with increasing PPP addition, from 72.3 in the control sample to 60.8 in the sample containing 15% PPP, indicating a significant darkening of the product. The a* parameter (redness) increased from 1.8 to 6.7, indicating a shift in color toward reddish-orange hues. Simultaneously, the b* parameter (yellowness) increased from 21.4 to 30.2, indicating an intensification of the yellow color of the pasta. Taken together, these changes indicate a shift from the light, creamy color of the control pasta to a darker, intensely orange color in the PPP-enriched samples.

The observed color changes are directly related to the presence of natural pigments in pumpkin peels, primarily carotenoids. The increase in the b* value, accompanied by an increase in the a* value, indicates a higher content of yellow-orange compounds, such as β-carotene and lutein [55,56]. A decrease in lightness (L*) is a typical effect of enriching cereal products with plant additives rich in dietary fiber and phenolic compounds. The darker color may result not only from the presence of pigments but also from non-enzymatic browning reactions occurring during drying or heat treatment [57,58].

From a technological perspective, color changes represent an important quality parameter, as pasta color is one of the key factors influencing consumer acceptance. Traditional pasta is characterized by a light yellow color, whereas enrichment with PPP results in a more intense orange hue, which may be perceived as visually appealing and associated with higher nutritional value. The literature emphasizes that carotenoid-rich additives can positively influence the perceived quality of a product, as consumers often associate a more intense color with a higher content of bioactive compounds [18,59]. However, excessive darkening of the product may also reduce consumer acceptance, especially among consumers accustomed to the traditional appearance of pasta.

The increase in the a* value may also be partially related to the presence of phenolic compounds, which can influence the product’s color, particularly after oxidation. Interactions between polyphenols and proteins may further modify color perception [22,60].

From a practical perspective, a PPP addition level of 5–10% may provide a beneficial visual effect by increasing color intensity without excessive darkening. At higher addition levels (15%), the color changes become much more pronounced and may require sensory evaluation to determine consumer acceptance.

### 2.8. Texture Profile Analysis (TPA) of Cooked Pasta

Texture profile analysis (TPA) of cooked pasta revealed a significant effect of pumpkin peel powder (PPP) on its mechanical properties (Table 8). Product hardness increased with increasing PPP content, from 4.8 N in the control sample to 6.6 N in the sample containing 15% PPP. In contrast, springiness showed a decreasing trend, declining from 0.91 to 0.84, indicating a reduced ability of the product to recover its original shape after deformation. A similar trend was observed for cohesiveness, which decreased from 0.62 to 0.55. Chewiness, which is a function of hardness, springiness, and cohesiveness, increased from 2.71 to 3.04, indicating that greater chewing effort was required for the PPP-enriched product. The increase in pasta hardness with increasing PPP addition can be explained by the presence of dietary fiber, which acts as a structural component reinforcing the product matrix.

Fiber particles may limit starch swelling during cooking and increase the mechanical resistance of the structure, resulting in a firmer and more compact product [28,61,62]. At the same time, the decrease in springiness and cohesiveness indicates a weakening of the gluten network. Replacing a portion of the wheat flour with plant material may lead to the dilution of gluten proteins, which are responsible for the elasticity and integrity of the pasta structure. As a result, the product becomes less elastic and more susceptible to mechanical damage [31,63].

The decrease in cohesiveness may also result from the heterogeneous structure introduced by PPP particles. Plant fibers may act as a dispersed phase, disrupting the continuity of the starch-protein matrix and leading to easier structural breakdown during chewing [22]. The increase in chewiness is a natural consequence of the increased product hardness despite the simultaneous decrease in springiness and cohesiveness. This indicates that pasta enriched with PPP requires greater chewing effort during consumption, which may influence sensory perception. In some cases, increased chewiness may be perceived positively as a characteristic feature of a more “whole-grain” or fiber-rich product.

From a technological perspective, the observed changes indicate a compromise between nutritional value and textural quality. Although increasing the fiber content improves the nutritional profile of the product, it also reduces its elastic properties and structural cohesiveness. Similar relationships have been reported in studies on pasta enriched with various sources of dietary fiber [25]. From a practical perspective, a PPP addition level of 5–10% appears to be the most beneficial, as it increases product hardness and functional value while causing only a moderate deterioration in structural parameters. Higher addition levels (15%) result in more pronounced changes that may require optimization of the formulation or processing conditions.

### 2.9. Starch Digestibility and Predicted Glycemic Index (pGI) of the Pasta with PPP

The addition of pumpkin peel powder (PPP) significantly affected the starch digestibility and predicted glycemic index (pGI) of the pasta (Table 9). The proportion of rapidly digestible starch (RDS) systematically decreased with increasing PPP addition, from 62.29% in the control sample to 48.47% in the sample containing 15% addition. Concurrently, the slowly digestible starch (SDS) fraction showed the opposite trend, increasing from 21.3% to 28.9%. The resistant starch (RS) content also increased from 6.2% to 10.8%, indicating beneficial changes from a nutritional perspective. As a consequence of these changes, the predicted glycemic index (pGI) decreased from 71.2 in the control sample to 58.7 at the highest PPP addition level.

The results clearly indicate that enriching pasta with PPP significantly improved the carbohydrate quality of the product. A decrease in the proportion of rapidly digestible starch (RDS) reduces the rapid release of glucose into the bloodstream after consumption, which is important for the prevention of metabolic diseases, such as type 2 diabetes [27,64,65]. The increase in the slowly digestible starch (SDS) fraction may be related to modifications in the structure of the starch–protein matrix in the presence of dietary fiber. Dietary fiber acts as a physical barrier, limiting the access of digestive enzymes to starch granules and thereby slowing the hydrolysis process [66].

A particularly beneficial effect is the increase in resistant starch (RS) content, which is not digested in the small intestine and functions similarly to dietary fiber. Resistant starch can be fermented in the large intestine, leading to the formation of short-chain fatty acids, which exert beneficial health effects, including supporting the gut microbiota [67]. The observed decrease in pGI is a direct consequence of changes in the starch profile. Products with a lower glycemic index are particularly desirable in the diets of individuals with disorders of carbohydrate metabolism and for obesity prevention. The literature emphasizes that enriching cereal products with dietary fiber and bioactive compounds is one of the most effective strategies for lowering their glycemic index [28].

The mechanism responsible for the reduction in pGI following PPP addition may be multifactorial. In addition to the physical barrier created by dietary fiber, phenolic compounds may also play a significant role, as they can inhibit the activity of digestive enzymes such as α-amylase and α-glucosidase [18]. Furthermore, changes in pasta structure resulting from increased water retention may affect the accessibility of starch to digestive enzymes. A more hydrated, yet less ordered, structure may promote the formation of starch complexes with other components, thereby limiting starch digestibility [31].

From a practical perspective, a PPP addition level of 10–15% appears to be the most beneficial, as it significantly reduces the pGI while simultaneously increasing the proportion of beneficial starch fractions. Such products can be classified as functional foods with the potential to support metabolic health.

However, it should be emphasized that the predicted glycemic index was calculated from an in vitro digestion model and should not be interpreted as equivalent to the glycemic response observed in human intervention studies.

### 2.10. Bioaccessibility of Phenolic Compounds (In Vitro Digestion)

Enrichment of pasta with pumpkin peel powder (PPP) significantly affected the bioaccessibility of phenolic compounds and antioxidant activity following simulated in vitro digestion (Table 10). The proportion of bioaccessible polyphenols increased gradually with increasing levels of PPP addition, from 48.2% in the control sample to 60.3% in the sample containing 15% addition. At the same time, a significant increase in antioxidant activity was observed after digestion. The values of this parameter increased more than fourfold, from 3.2 to 14.6 µmol TE/g, with increasing PPP levels. These results indicate that not only does the total content of bioactive compounds increase (as shown previously), but also the fraction potentially available for absorption by the body after digestion. The increased bioaccessibility of polyphenols following PPP addition indicates that pumpkin peels are not only a rich source of these compounds but also provide a matrix that facilitates their release during digestion.

The literature emphasizes that the food matrix and interactions among its components play a key role in the bioaccessibility of phenolic compounds [68]. One possible mechanism underlying the increased bioaccessibility is the partial release of fiber-bound polyphenols during enzymatic digestion. In plant-based foods, a significant proportion of phenolic compounds is present in a bound form (e.g., associated with cell wall polysaccharides), which limits their bioaccessibility. The digestion process may lead to their gradual release, thereby increasing their potential bioaccessibility [22,69,70]. The observed increase in antioxidant activity following digestion is also noteworthy. This finding suggests that digestion products may exhibit greater activity than the compounds present in the undigested product. This phenomenon may result from the formation of metabolites with stronger antioxidant properties or from synergistic interactions among the released compounds [71,72]. The increase in antioxidant activity after digestion is particularly important from a physiological perspective, as only compounds that become available following passage through the gastrointestinal tract can exert biological effects. These results indicate that PPP-enriched pasta may serve as an effective dietary source of bioactive antioxidants. It is also worth noting that dietary fiber may play a dual role. On the one hand, it may limit the immediate bioaccessibility of certain compounds by binding them, while on the other hand, it enables their gradual release during the later stages of digestion, potentially prolonging their biological activity [68,73]. From a technological perspective, these results confirm that the use of by-products, such as pumpkin peels, not only increases the content of bioactive compounds in food but also enhances their biological functionality. This is consistent with current trends in the development of functional foods with enhanced nutrient bioaccessibility [9,74].

Although the increased bioaccessibility observed in vitro is encouraging, gastrointestinal physiology is considerably more complex than simulated digestion models. Consequently, the present findings should be regarded as an indication of potential nutritional benefits rather than direct evidence of improved bioavailability in humans.

### 2.11. Oxidative Stability During Storage of Pasta Enriched with PPP (30 Days)

The results presented in Table 11 indicate a clear effect of PPP addition on the oxidative stability of pasta during 30 days of storage. Both the peroxide value (PV) and the TBARS index decreased progressively with increasing PPP content, suggesting its antioxidant potential. For the peroxide value (PV), which reflects the initial stage of lipid oxidation, a gradual decrease was observed from 3.8 meq O_2_/kg fat in the control sample (0% PPP) to 2.3 meq O_2_/kg fat in the sample containing 15% PPP. This indicates that the presence of PPP effectively limits the formation of primary lipid oxidation products. A similar trend was observed for TBARS values, which are indicative of secondary lipid oxidation products, such as malondialdehyde (MDA). These values decreased from 0.82 mg MDA/kg in the control sample to 0.55 mg MDA/kg in the sample containing the highest level of PPP. The observed effect can be explained by the presence of bioactive compounds in PPP, particularly polyphenols, which are capable of neutralizing free radicals and interrupting the chain reactions involved in lipid oxidation.

Similar findings have been reported in the literature, where plant-derived additives rich in antioxidants effectively improved the oxidative stability of lipid-containing and meat products [75,76]. The results also indicate a dose-dependent effect, as increasing the proportion of PPP resulted in a greater reduction in oxidative processes. This may have important practical implications, suggesting that PPP could be used as a natural functional ingredient to extend the shelf life of food products. At the same time, it should be noted that excessive levels of PPP may affect other product characteristics, such as taste or texture, highlighting the need for further optimization studies.

### 2.12. Prebiotic Potential (In Vitro)

The results presented in Table 12 indicate that the addition of PPP significantly affects the in vitro prebiotic potential of the tested samples. The growth of both *Lactobacillus* and *Bifidobacterium* increased with increasing PPP levels, suggesting a beneficial effect on the development of the intestinal microbiota. For *Lactobacillus*, a systematic increase in cell counts was observed, from 7.2 log CFU/mL in the control sample to 8.7 log CFU/mL in the sample containing 15% PPP. A similar trend was observed for *Bifidobacterium*, with cell counts increasing from 7.0 to 8.5 log CFU/mL. This increase indicates a clear stimulatory effect that intensified with increasing PPP content in the product. The observed phenomenon can be explained by the presence of prebiotic components in PPP, such as dietary fiber and indigestible oligosaccharides. These compounds are not digested in the upper gastrointestinal tract but instead serve as substrates for beneficial intestinal bacteria, thereby promoting their growth. Similar findings have been reported in the literature, where plant-derived ingredients rich in dietary fiber demonstrated the ability to selectively stimulate the growth of probiotic bacteria [77,78].

The obtained results also indicate a dose-dependent effect, as higher concentrations of PPP promoted greater growth of the tested microorganisms. This finding may be important for the development of functional foods aimed at improving gut health. However, it should be noted that the in vitro results require confirmation in in vivo studies, where additional physiological factors may influence the actual prebiotic activity of PPP.

### 2.13. Multivariate Statistical Analysis of PPP-Enriched Pasta

**Figure 1:**
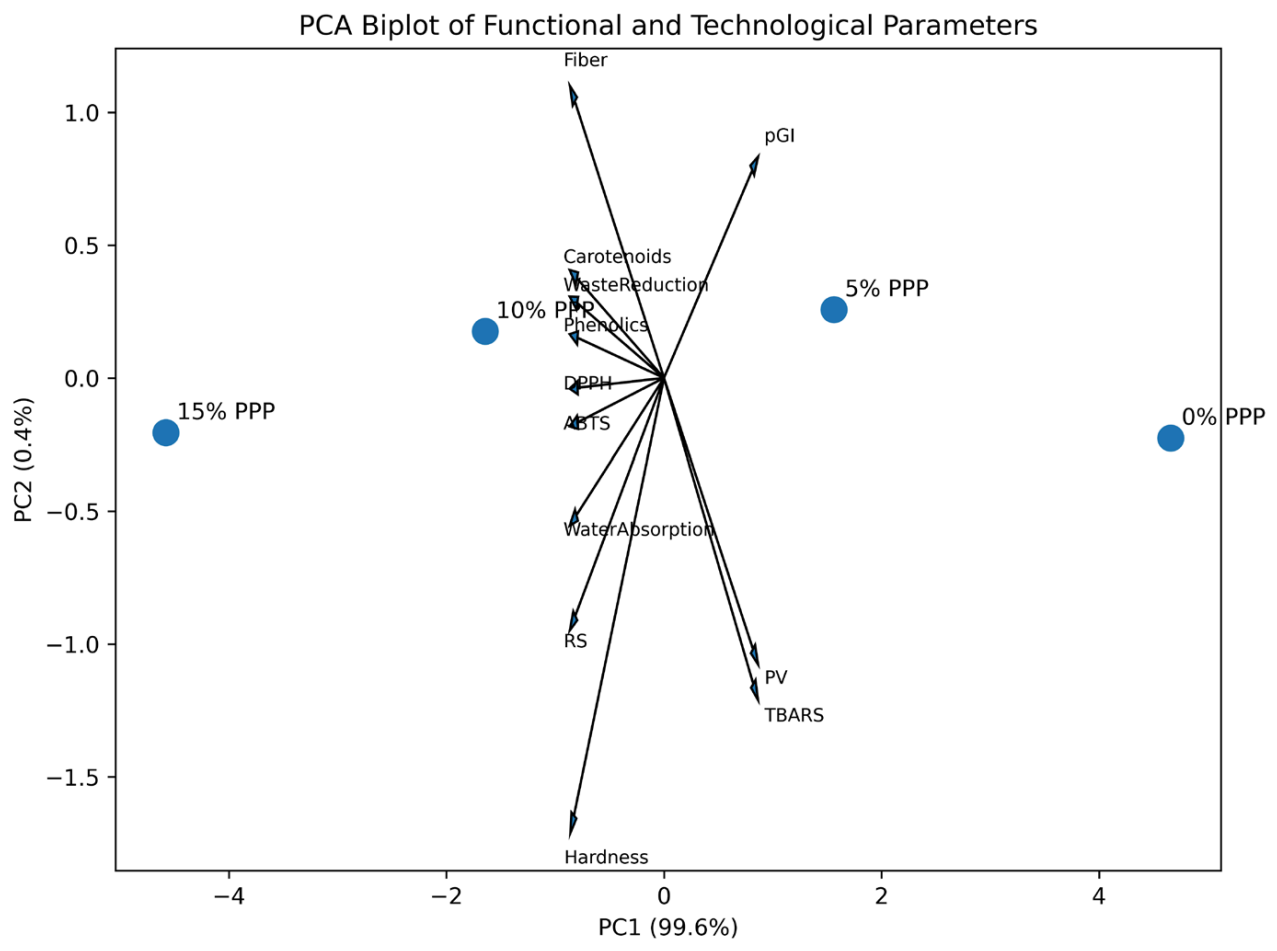
Principal Component Analysis (PCA) biplot showing the multivariate differentiation of pasta samples enriched with pumpkin peel powder (PPP) based on nutritional, technological, antioxidant, rheological, and environmental parameters.

To obtain a more comprehensive understanding of the influence of pumpkin peel powder (PPP) on pasta quality, advanced multivariate statistical analyses were performed, including principal component analysis (PCA), correlation analysis, hierarchical cluster analysis, and heatmap visualization. These approaches enabled the simultaneous evaluation of technological, nutritional, antioxidant, rheological and prebiotic variables. Principal Component Analysis revealed a possible reason separation of pasta samples according to the level of PPP enrichment (Figure 1).

The first principal component (PC1) explained the majority of the total variance and was associated with variables related to functional quality, including total dietary fiber, phenolic compounds, carotenoids, antioxidant activity (DPPH and ABTS), resistant starch (RS), and prebiotic potential. Samples containing higher levels of PPP were positioned on the positive side of PC1, indicating a contribution of bioactive compounds and health-promoting properties. In contrast, the control sample was associated with a higher predicted glycemic index (pGI), rapidly digestible starch (RDS), and higher lightness (L*) values, reflecting its lower functional value.

The PCA model also demonstrated that the incorporation of PPP induced coordinated modifications across multiple quality attributes rather than isolated compositional changes. Particularly strong associations were observed between antioxidant parameters and dietary fiber content, suggesting that PPP acts as a multifunctional ingredient simultaneously improving the nutritional and technological characteristics of pasta. Moreover, samples enriched with 10–15% PPP occupied the most favorable region of the PCA plot, indicating an optimal balance between enhanced bioactivity and acceptable technological performance (Figure 1).

It should be noted, however, that PCA identifies associations among variables rather than causal relationships. Therefore, the observed clustering supports the overall consistency of the experimental results but should not be interpreted as evidence of direct mechanistic interactions between the analyzed parameters.

**Figure 2:**
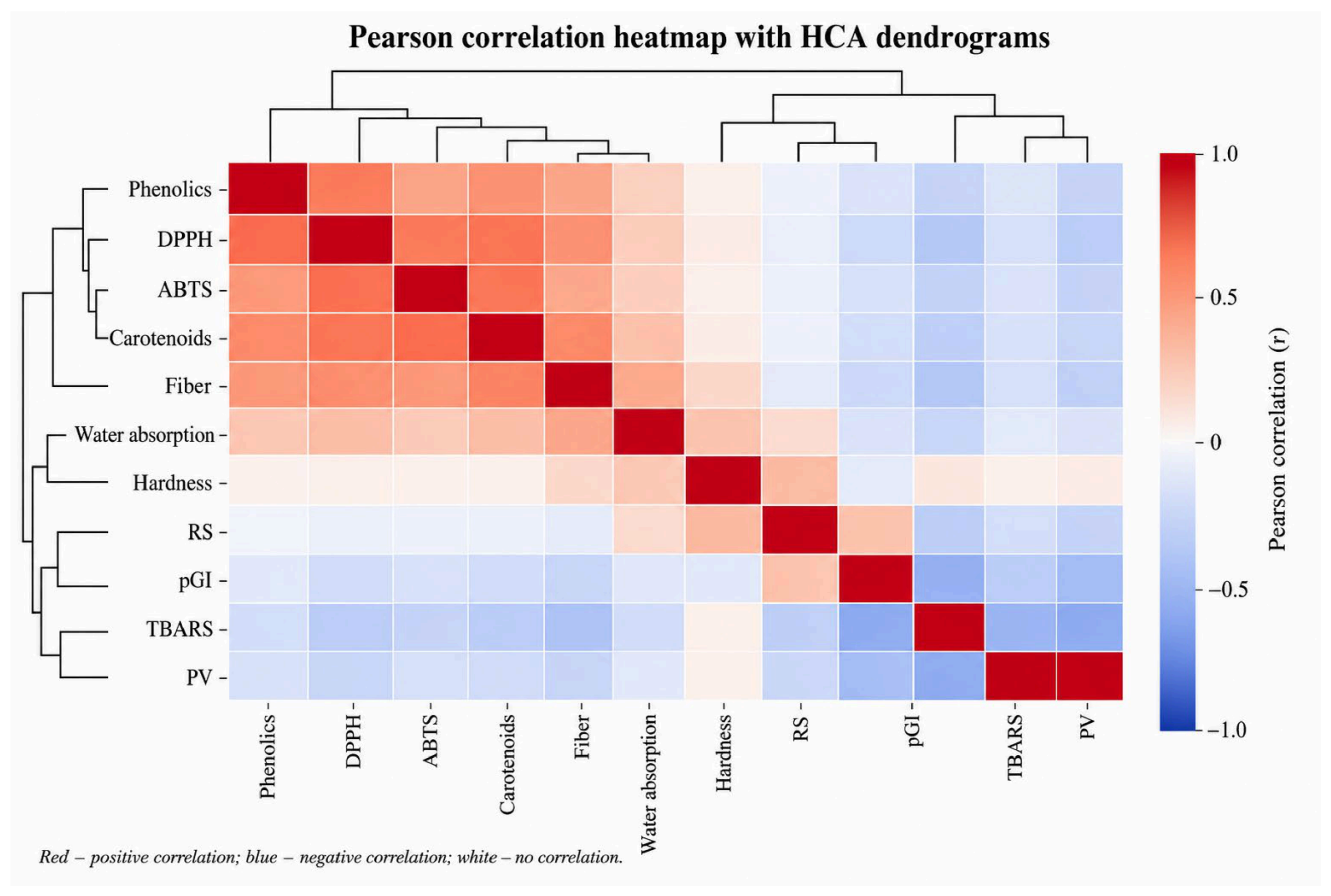
Pearson correlation heatmap with HCA dendrograms.

The correlation heatmap combined with hierarchical cluster analysis (HCA) revealed distinct relationships among the technological, nutritional, and antioxidant properties of pasta enriched with pumpkin peel powder (PPP). The clustering pattern separated the analyzed variables into two major groups, reflecting their biological and technological interdependence (Figure 2). The first cluster included dietary fiber content, phenolic compounds, carotenoids, antioxidant activity (DPPH and ABTS), resistant starch (RS), water absorption, and hardness. These variables showed predominantly positive correlations, indicating that increasing PPP incorporation simultaneously enhanced the concentration of bioactive compounds and several functional properties of the product. The close association among dietary fiber, phenolic compounds, carotenoids, and antioxidant indicators suggests that pumpkin peel powder acted as a major source of antioxidant constituents, thereby contributing to the overall functional quality of the pasta. Within this cluster, resistant starch and water absorption were also positively associated with fiber-rich formulations. This observation is consistent with the ability of dietary fiber to modify the starch–protein matrix and increase water-binding capacity. Furthermore, the moderate association between hardness and fiber-related parameters suggests that structural reinforcement of the pasta matrix occurred as the PPP concentration increased.

A second cluster comprised pGI, peroxide value (PV), and TBARS. These variables exhibited inverse relationships with the bioactive and nutritional parameters grouped in the first cluster. The negative association between pGI and both dietary fiber and resistant starch indicates that PPP enrichment contributed to a slower starch digestion profile and a lower predicted glycemic response. Similarly, the negative correlations observed between antioxidant-related variables and oxidative stability indicators (PV and TBARS) suggest that the phenolic compounds and carotenoids present in PPP effectively limited lipid oxidation processes. The dendrogram structure further demonstrated that DPPH and ABTS were closely associated, reflecting their common assessment of antioxidant capacity. Likewise, PV and TBARS formed a separate subcluster, confirming that both indicators represent related stages of lipid oxidative deterioration. Dietary fiber, phenolic compounds, and carotenoids were grouped in close proximity, highlighting the role of pumpkin peel constituents as key drivers of the observed improvements in functional quality.

Overall, the combined correlation and clustering analyses indicate that the incorporation of pumpkin peel powder resulted in coordinated improvements in nutritional value and antioxidant potential while simultaneously reducing glycemic potential and oxidative deterioration. These findings support the suitability of PPP as a multifunctional ingredient for the development of value-added pasta products.

Overall, the multivariate statistical approach substantially strengthened the interpretation of the obtained results by demonstrating that PPP enrichment induces systematic and interconnected modifications across nutritional, technological, antioxidant and microbiological dimensions. The integration of PCA, hierarchical cluster analysis and heatmap analysis clearly supports the classification of PPP-enriched pasta as a sustainable functional food aligned with current circular economy strategies and modern nutritional recommendations.

## 3. Materials and Methods

### 3.1. Research Material

The experimental material consisted of pumpkin peels obtained from mature fruits of *Cucurbita maxima* generated as a by-product during pumpkin puree production. The raw material was collected from a local agricultural producer immediately after technological processing. The peels were washed with distilled water to remove impurities and subsequently dried in a laboratory convective dryer (IKA-Werke GmbH & Co. KG, Staufen, Germany) at 50 ± 2 °C until constant weight was achieved, according to the gravimetric drying procedure described by the AOAC International [79].

The dried material was ground using a laboratory mill (IKA-Werke GmbH & Co. KG, Staufen, Germany) and sieved through a 0.5-mm stainless-steel sieve. The resulting pumpkin peel powder (PPP) was stored in airtight polyethylene containers protected from light and moisture at 4 °C until further analyses. The particle size of PPP was controlled by sieve analysis. All of particles were smaller than 500 μm, ensuring homogeneous incorporation into the pasta matrix. Future studies should include laser diffraction analysis to determine the complete particle-size distribution.

### 3.2. Chemicals and Reagents

All chemicals and reagents used in the study were of analytical grade. Folin–Ciocalteu reagent, methanol, acetone, hexane, hydrochloric acid, Trolox, and gallic acid standards were purchased from certified suppliers. Spectrophotometric analyses were performed using a UV–Vis spectrophotometer (Shimadzu, Kyoto, Japan), while chromatographic analyses were conducted using a high-performance liquid chromatography system (HPLC) (Shimadzu, Kyoto, Japan).

### 3.3. Proximate Chemical Composition

Moisture content was determined using the oven-drying method according to the procedure of AOAC International [79]. Ash content was analyzed by incineration in a muffle furnace (Nabertherm GmbH, Lillenthal, Germany) at 550 °C [80]. Crude protein content was determined using the Kjeldahl method with a nitrogen conversion factor of 6.25, following the AOCA 928.08 standard [81]. Crude fat content was measured by Soxhlet extraction using petroleum ether as solvent [82].

Total dietary fiber, including soluble and insoluble fractions, was determined using the enzymatic–gravimetric method described by Elleuch et al. [22]. All analyses were performed in triplicate.

Total carbohydrate content was calculated by difference using the following equation:

Total carbohydrates (g/100 g) = 100 − (moisture + protein + fat + ash + dietary fiber)

### 3.4. Preparation of Pasta Samples

Four pasta formulations were prepared containing 0 (control), 5, 10 and 15% (*w*/*w*) pumpkin peel powder (PPP) as a partial replacement of wheat flour (type 500) on a weight basis.

Dry ingredients were mixed for 10 min in a laboratory mixer before water addition. Water was gradually incorporated until dough moisture reached approximately 32%. Dough was kneaded for 15 min and then extruded using a laboratory pasta extruder equipped with a spaghetti die. Fresh pasta strands were dried under controlled conditions at 40 °C until moisture content was below 12%. To ensure biological replication, three independent pasta batches were produced for each formulation on separate production days. Each analytical determination was subsequently performed in triplicate for every batch, providing three biological replicates and three analytical replicates. Dried pasta samples were sealed in polyethylene bags and stored at ambient temperature until further analyses.

### 3.5. Functional Properties

Water-holding capacity (WHC), oil-holding capacity (OHC), and swelling capacity were determined according to the procedures described by Elleuch et al. [22]. Emulsion stability and emulsifying properties were additionally evaluated to determine the technological suitability of pumpkin peel powder for food applications.

Water-Holding Capacity (WHC): One gram of PPP was mixed with 20 mL distilled water and maintained at room temperature for 24 h with intermittent mixing. Samples were centrifuged at 4000× *g* for 20 min and the supernatant was discarded. WHC was calculated as grams of retained water per gram of dry sample.

Oil-Holding Capacity (OHC): The procedure described for WHC was repeated using refined sunflower oil instead of water. OHC was expressed as grams of retained oil per gram of dry sample.

Swelling Capacity: One gram of PPP was transferred into a graduated cylinder containing 10 mL distilled water and left undisturbed for 24 h. Swelling capacity was calculated as the increase in sample volume (mL g^−1^), while the swelling index was expressed as the percentage increase in volume relative to the initial volume.

Emulsion Stability: PPP (2 g) was homogenized with 20 mL distilled water and 20 mL sunflower oil using a laboratory homogenizer (Revvity, Inc. Watham, MA, USA) operating at 10,000 rpm for 2 min. After centrifugation, emulsion stability was calculated as the percentage of the emulsified layer remaining after heating at 80 °C for 30 min followed by cooling.

All determinations were performed in triplicate.

### 3.6. Determination of Phenolic Compounds and Antioxidant Activity

Total phenolic content (TPC) was determined using the Folin–Ciocalteu colorimetric method according to Singleton and Rossi [83] with slight modifications. Absorbance was measured at 765 nm and results were expressed as mg gallic acid equivalents (GAE)/g dry matter.

Briefly, 1 g of sample was extracted with 80% methanol under continuous shaking for 60 min at room temperature. The extracts were centrifuged at 5000× *g* for 10 min and the supernatants were collected. Aliquots of the extract (0.5 mL) were mixed with Folin–Ciocalteu reagent followed by sodium carbonate solution. After incubation for 30 min in darkness, absorbance was measured at 765 nm using a UV–Vis spectrophotometer (UV- 2600i, Shimadzu, Japan). Gallic acid was used as the calibration standard and results were expressed as mg gallic acid equivalents (GAE) per gram of dry matter.

To avoid discrepancies between raw materials and enriched pasta, all phenolic results were expressed on the same dry-matter basis using identical extraction procedures for every sample.

#### 3.6.1. Determination of Total Carotenoids

Total carotenoid content was determined spectrophotometrically following the procedure described by Lichtenthaler and Buschmann [84], with slight modifications. The samples were homogenized in 80% (*v*/*v*) acetone using a sample-to-solvent ratio of 1:20 (*m*/*v*). Carotenoids were extracted using an acetone–hexane solvent system protected from direct light and the extraction was carried out in the dark for 20 min at 4 °C. After centrifugation (5000× *g* for 10 min), the clear supernatant was transferred into a quartz cuvette. Absorbance was measured at 470 nm, with 80% acetone used as the blank solution. Total carotenoid concentration was calculated from β-carotene calibration curves and expressed as mg β-carotene equivalents per 100 g dry matter. All analyses were performed in triplicate.

#### 3.6.2. Antioxidant Activity

The antioxidant activity of pumpkin peel powder (PPP) and pasta extracts was deter-mined using both the DPPH and ABTS radical scavenging assays. Methanolic extracts prepared for total phenolic determination were used for both analyses. Antioxidant activity was evaluated using DPPH radical scavenging assay according to Brand-Williams et al. [85] and ABTS assay according to Re et al. [41].

DPPH Radical Scavenging Assay: The free radical scavenging capacity was determined using the stable 2,2-diphenyl-1-picrylhydrazyl (DPPH·) radical. A freshly prepared DPPH methanolic solution (0.1 mM) was mixed with the sample extract at a ratio of 3.9:0.1 (*v*/*v*). The reaction mixture was incubated in darkness at room temperature for 30 min before absorbance measurement at 517 nm using a UV–Vis spectrophotometer. Trolox was used as the reference antioxidant for calibration (0–500 μM). Antioxidant activity was expressed as μmol trolox equivalents (TE) per gram of dry matter.

ABTS Radical Cation Assay: The ABTS radical cation (ABTS·+) was generated by reacting 7 mM ABTS solution with 2.45 mM potassium persulfate and allowing the mixture to stand in darkness for 16 h at room temperature. Before analysis, the radical solution was diluted with phosphate-buffered saline to obtain an absorbance of 0.70 ± 0.02 at 734 nm. A volume of 100 μL of extract was mixed with 3.0 mL of diluted ABTS solution and incubated for 6 min. Absorbance was measured at 734 nm. Results were calculated using a trolox calibration curve and expressed as μmol TE g^−1^ dry matter.

All antioxidant measurements were performed in triplicate using three independent biological replicates.

### 3.7. Cooking Quality of Pasta

The optimum cooking time was determined by periodically pressing cooked pasta strands between two glass plates until the white ungelatinized core disappeared. Cooking loss was determined by evaporating the cooking water to dryness and expressing the residue as a percentage of the initial dry pasta weight. Water absorption was calculated as the percentage increase in pasta weight after cooking according to the following equation:

Water absorption (%) = (Weight after cooking − Initial weight)/Initial weight) × 100

Each analysis was conducted on three independently prepared pasta batches with analytical triplicates.

### 3.8. Technological and Texture Analysis

Texture profile analysis was performed on cooked pasta samples using a TA.XTplus Texture Analyzer (Stable Micro Systems Ltd., Surrey, UK) equipped with a cylindrical compression probe.

Cooked pasta strands were equilibrated to room temperature before analysis. Samples were subjected to a double-compression test to 50% deformation at a crosshead speed of 1.0 mm s^−1^ with a 5 s interval between compressions. The following parameters were calculated automatically by the instrument software (Exponent 6.2): hardness, springiness, cohesiveness, and chewiness. Ten individual pasta strands from each biological replicate were analysed [86].

### 3.9. Rheological Properties

The rheological behavior of pasta dough was evaluated using a rotational rheometer. Storage modulus (G′), loss modulus (G″), and apparent viscosity were measured as a function of oscillatory frequency according to the rheological approach described by Dobraszczyk and Morgenstern [87].

The viscoelastic properties of fresh pasta dough were evaluated using a controlled-stress rotational rheometer (Netzsch Group, Selb, Germany) equipped with a parallel-plate geometry (40 mm diameter; 1 mm gap). Prior to frequency sweep measurements, strain sweep tests (0.01–100%) were carried out to determine the linear viscoelastic region (LVR). All subsequent measurements were performed within the LVR at a strain of 0.5%. Frequency sweep measurements were conducted over the range of 0.1–10 Hz at 25 °C. The storage modulus (G′), loss modulus (G″), and complex viscosity (η*) were recorded automatically using the rheometer software (rSpace 2.2).

Each measurement was repeated for three independently prepared dough batches.

### 3.10. Color Measurement

Color parameters (L*, a*, b*) were measured using a colorimeter in the CIE Lab system according to the methodology reported by Pathare et al. [88]. Colour parameters were measured using a calibrated Minolta CR-400 colorimeter operating in the CIE Lab* colour space. Measurements were performed under standard illuminant D65 with a 10° observer angle. For each sample, ten independent measurements were carried out at different locations on the pasta surface. The recorded parameters included: L* (lightness), a* (red-green coordinate), and b* (yellow-blue coordinate). The average values were used for statistical analysis.

### 3.11. Oxidative Stability

Lipid oxidation was evaluated by determining peroxide value (PV) and thiobarbituric acid reactive substances (TBARS) according to the methodology described by Shahidi and Zhong [76]. Measurements were conducted during storage under controlled conditions.

Oxidative stability was evaluated during storage of dried pasta for 30 days.

Samples were stored in polyethylene bags protected from direct light at 25 ± 2 °C and relative humidity of approximately 55%. Lipid oxidation was evaluated by determining peroxide value (PV) and thiobarbituric acid reactive substances (TBARS). Peroxide value was determined according to the AOCS official method and expressed as meq O_2_ kg^−1^ fat.

TBARS values were determined spectrophotometrically at 532 nm and expressed as mg malondialdehyde (MDA) kg^−1^ sample using a calibration curve prepared with 1,1,3,3-tetraethoxypropane. Measurements were performed on day 0 and after 30 days of storage.

### 3.12. In Vitro Starch Digestibility and Predicted Glycemic Index

Rapidly digestible starch (RDS), slowly digestible starch (SDS), and resistant starch (RS) fractions were determined using the enzymatic procedure developed by Englyst et al. [64]. Based on these results, the predicted glycemic index (pGI) was calculated.

Starch digestibility was determined using a standardized in vitro enzymatic digestion model simulating gastrointestinal conditions. Ground cooked pasta samples were incubated with pancreatic α-amylase and amyloglucosidase under controlled temperature (37 °C) and pH conditions. Rapidly digestible starch (RDS), slowly digestible starch (SDS) and resistant starch (RS) fractions were calculated according to the glucose released after predetermined digestion times. The predicted glycaemic index (pGI) was calculated using the hydrolysis index obtained from starch digestion kinetics according to previously validated equations.

All analyses were carried out using three independent biological replicates and analytical triplicates.

### 3.13. In Vitro Digestion and Bioaccessibility

The bioaccessibility of phenolic compounds was assessed using a standardized simulated gastrointestinal digestion model including oral, gastric, and intestinal phases according to Minekus et al. [72]. Following digestion, antioxidant activity and phenolic content were analyzed in the bioaccessible fraction.

The bioaccessibility of phenolic compounds was evaluated using a standardized static in vitro gastrointestinal digestion model based on the INFOGEST protocol with slight modifications.

Cooked pasta samples were freeze-dried, ground and accurately weighed (5 g). Simulated oral digestion was initiated by mixing the samples with simulated salivary fluid containing α-amylase (75 U mL^−1^) at pH 7.0 for 2 min at 37 °C.

The gastric phase was performed by adding simulated gastric fluid containing pepsin (2000 U mL^−1^). The pH was adjusted to 3.0 using 1 M HCl and samples were incubated for 2 h at 37 °C under continuous agitation (100 rpm).

The intestinal phase was initiated by adding simulated intestinal fluid containing pancreatin (100 U mL^−1^ based on trypsin activity) and bile salts (10 mM). The pH was adjusted to 7.0 using 1 M NaOH and incubation continued for another 2 h at 37 °C.

Following digestion, samples were centrifuged at 6000× *g* for 20 min. The supernatants were collected as the bioaccessible fraction.

Total phenolic compounds and antioxidant activity (DPPH and ABTS assays) were determined in the bioaccessible fraction using the procedures described above.

Bioaccessibility (%) was calculated as:

Bioaccessibility (%) = (Phenolics in the digested fraction/Phenolics in the undigested sample) × 100

### 3.14. In Vitro Prebiotic Activity

The prebiotic activity of PPP-enriched pasta was evaluated in vitro by monitoring the growth of probiotic bacteria belonging to Lactobacillus and Bifidobacterium genera according to the methodology proposed by Gibson et al. [78]. Bacterial growth was expressed as log CFU/mL.

The prebiotic potential of PPP-enriched pasta was evaluated using representative probiotic bacterial strains including *Lactobacillus plantarum* ATCC 14917 and *Bifidobacterium animalis* subsp. *lactis* BB-12. Lyophilized pasta samples were sterilized by UV irradiation before fermentation experiments. Basal MRS medium without glucose was supplemented with 1% (*w*/*v*) sample powder as the sole carbon source. Glucose-containing medium served as the positive control, whereas carbohydrate-free medium served as the negative control. The bacterial inoculum (approximately 10^6^ CFU mL^−1^) was incubated anaerobically at 37 °C for 24 h. Bacterial growth was determined by plate counting on selective agar media and expressed as log CFU mL^−1^.

Each experiment was carried out in triplicate using three independent biological replicates.

### 3.15. Statistical Analysis

All experimental results are presented as mean ± standard deviation (SD).

Three independently produced pasta batches were prepared for each formulation (biological replicates), and each analytical measurement was performed in triplicate (analytical replicates).

Prior to statistical analysis, data normality was verified using the Shapiro–Wilk test, while homogeneity of variances was assessed using Levene’s test.

Differences among formulations were evaluated by one-way analysis of variance (ANOVA).

When significant differences were detected (*p* < 0.05), Tukey’s honestly significant difference (HSD) test was applied for multiple comparisons.

Pearson correlation coefficients were calculated to evaluate relationships among technological, nutritional, antioxidant and functional parameters.

Principal Component Analysis (PCA) was performed as an exploratory multivariate technique to visualize relationships among samples and measured variables. Owing to the limited number of experimental groups, PCA was interpreted descriptively and was not used for inferential statistical conclusions.

Hierarchical Cluster Analysis (HCA) using Ward’s method and Euclidean distance was performed to identify similarities among variables.

All statistical analyses were carried out using Statistica 13.3 software (TIBCO Software Inc., Palo Alto, CA, USA). Statistical significance was accepted at *p* < 0.05.

## 4. Conclusions

The present study demonstrated that pumpkin peels, generated as a by-product of pumpkin processing, represent a valuable source of dietary fiber and bioactive compounds with significant technological and nutritional potential.

Enrichment of pasta with pumpkin peel powder (PPP) significantly increased the contents of total dietary fiber, phenolic compounds, carotenoids, and antioxidant activity. Moreover, PPP favorably modified starch digestibility by increasing resistant starch content and reducing the predicted glycemic index, which may contribute to an improved metabolic response. The observed enhancement in oxidative stability and prebiotic potential further indicates that pumpkin peel powder may support both extended product shelf life and gut health.

From a technological perspective, PPP affected the rheological properties, cooking quality, texture, and color parameters of pasta. Higher levels of PPP increased water absorption, dough viscosity, hardness, and color intensity while simultaneously contributing to moderate weakening of the gluten network and increased cooking losses. Nevertheless, the obtained values remained within acceptable technological ranges for pasta products.

Multivariate statistical analyses confirmed a dose-dependent relationship between PPP incorporation and the functional quality of the final products. Samples enriched with 10–15% PPP exhibited the greatest improvements in antioxidant and nutritional properties. However, considering the balance between functional enhancement and technological quality, the 10% enrichment level appeared to provide the most favorable compromise.

Overall, the findings indicate that pumpkin peel powder can serve as an effective functional ingredient and sustainable raw material for the development of innovative cereal products with improved nutritional value. Future studies should focus on in vivo evaluation of bioavailability, consumer acceptance, shelf-life stability, environmental performance, and the industrial-scale application of pumpkin peel-enriched foods.

Despite these promising results, further studies involving sensory evaluation, extended storage experiments under commercial conditions, and human intervention trials are necessary before pumpkin peel powder-enriched pasta can be recommended for large-scale commercial application.

## Figures and Tables

**Table 1:** Chemical composition of pumpkin peel powder (PPP).

Factors	Value
Dry matter (%)	92.4 ± 0.3
Protein (%)	8.7 ± 0.2
Fat (%)	3.1 ± 0.1
Ash (%)	6.5 ± 0.2
Total fiber (%)	42.8 ± 1.1
Total carbohydrate content	31.3 ± 0.4
Polyphenols (mg GAE/g d.m.)	18.6 ± 0.9
Carotenoids (mg/100 g d.m.)	21.3 ± 1.2

**Table 2:** Functional properties of pumpkin peel powder (PPP).

Factors	Value
WHC (g water/g sample)	4.82 ± 0.21
OHC (g oil/g sample)	2.36 ± 0.14
Swelling index	5.9 ± 0.3
Swelling capacity (mL/g)	41.2 ± 1.8
Emulsion stability (%)	38.5 ± 1.6

**Table 3:** Change in the composition of pasta enriched with pumpkin peel powder (PPP).

Factors	0%	5% PPP	10% PPP	15% PPP
Dry matter (%)	88.28 ± 0.04 a	88.12 ± 0.02 a	87.85 ± 0.03 a	87.72 ± 0.06 a
Protein (g/100 g d.m.)	12.34 ± 0.03 a	11.93 ± 0.02 b	11.49 ± 0.04 c	11.21 ± 0.02 d
Fat (g/100 g d.m.)	1.48 ± 0.02 d	1.57 ± 0.02 c	1.73 ± 0.02 b	1.74 ± 0.02 a
Ash (g/100 g d.m.)	0.95 ± 0.02 d	1.17 ± 0.02 c	1.24 ± 0.02 b	1.32 ± 0.01 a
Total dietary fiber (g/100 g d.m.)	3.33 ± 0.02 d	5.57 ± 0.03 c	7.40 ± 0.07 b	8.89 ± 0.04 a
Insoluble fiber (g/100 g)	2.43 ± 0.02 d	4.52 ± 0.03 c	5.85 ± 0.03 b	6.83± 0.04 a
Soluble fiber (g/100 g)	0.9 ± 0.01 d	1.05 ± 0.01 c	1.55 ± 0.01 b	2.06 ± 0.03 a
Total carbohydrate content	70.18 ± 0.03 a	67.88 ± 0.02 b	65.99 ± 0.04 c	64.56 ± 0.03 d

Abbreviations: PPP—pumpkin peel powder. Values are expressed as mean ± standard deviation. Different superscript letters (a–d) within the same row indicate significant differences between means (*p* < 0.05).

**Table 4:** Bioactive compounds and antioxidant activity.

Factors	0%	5% PPP	10% PPP	15% PPP
Total phenolics (mg GAE/g d.m.)	2.50 ± 0.05 d	3.31 ± 0.04 c	4.11 ± 0.04 b	4.92 ± 0.04 a
Total carotenoids (mg/100 g)	0.47 ± 0.05 d	1.51 ± 0.03 c	2.55 ± 0.03 b	3.59 ± 0.04 a
DPPH (µmol TE/g)	4.87 ± 0.04 d	9.75 ± 0.02 c	15.32 ± 0.04 b	19.94 ± 0.04 a
ABTS (µmol TE/g)	5.40 ± 0.04 d	10.91 ± 0.03 c	17.23 ± 0.06 b	22.73 ± 0.04 a

Abbreviations: PPP—pumpkin peel powder. Values are expressed as mean ± standard deviation. Different superscript letters (a–d) within the same row indicate significant differences between means (*p* < 0.05).

**Table 5:** Cooking and technological properties of pasta.

Factors	0%	5% PPP	10% PPP	15% PPP
Cooking time (min)	9.5 ± 0.2 a	9.3 ± 0.2 a	9.0 ± 0.3 b	8.8 ± 0.2 c
Cooking loss (%)	5.1 ± 0.3 d	5.9 ± 0.2 c	6.8 ± 0.3 b	7.8 ± 0.4 a
Water absorption (%)	145 ± 3 d	152 ± 4 c	160 ± 5 b	168 ± 4 a

Abbreviations: PPP—pumpkin peel powder. Values are expressed as mean ± standard deviation. Different superscript letters (a–d) within the same row indicate significant differences between means (*p* < 0.05).

**Table 6:** Rheological properties of pasta dough.

Factors	0%	5% PPP	10% PPP	15% PPP
G′ (Pa)	1450 ± 60 d	1590 ± 70 c	1720 ± 80 b	1890 ± 90 a
G″ (Pa)	420 ± 25 d	460 ± 30 c	510 ± 35 b	580 ± 40 a
Viscosity (Pa·s)	38.5 ± 1.8 d	41.2 ± 2.0 c	45.6 ± 2.2 b	50.3 ± 2.5 a

Abbreviations: PPP, pumpkin peel powder; G′, storage modulus (elastic modulus); G″, loss modulus (viscous modulus); Pa, pascal; Pa·s, pascal-second. Values are expressed as mean ± standard deviation. Different superscript letters (a–d) within the same row indicate significant differences between means (*p* < 0.05).

**Table 7:** Color parameters (CIE Lab*).

Factors	0%	5% PPP	10% PPP	15% PPP
L*	72.3 ± 1.1 a	68.5 ± 1.0 b	64.2 ± 1.2 c	60.8 ± 1.3 d
a*	1.8 ± 0.2 d	3.6 ± 0.3 c	5.1 ± 0.3 b	6.7 ± 0.4 a
b*	21.4 ± 0.8 d	24.8 ± 0.9 c	27.6 ± 1.0 b	30.2 ± 1.1 a

Abbreviations: PPP, pumpkin peel powder; L*, lightness; a*, red–green coordinate; b*, yellow–blue coordinate. Values are expressed as mean ± standard deviation. Different superscript letters (a–d) within the same row indicate significant differences between means (*p* < 0.05).

**Table 8:** Texture profile analysis (TPA) of cooked pasta.

Factors	0%	5% PPP	10% PPP	15% PPP
Hardness (N)	4.8 ± 0.3 d	5.2 ± 0.2 c	5.9 ± 0.3 b	6.6 ± 0.4 a
Springiness	0.91 ± 0.02 a	0.89 ± 0.02 a	0.87 ± 0.03 b	0.84 ± 0.02 c
Cohesiveness	0.62 ± 0.03 a	0.60 ± 0.03 a	0.58 ± 0.02 b	0.55 ± 0.03 c
Chewiness	2.71 ± 0.2 c	2.78 ± 0.2 b	2.98 ± 0.2 a	3.04 ± 0.3 a

Abbreviations: PPP—pumpkin peel powder. Values are expressed as mean ± standard deviation. Different superscript letters (a–d) within the same row indicate significant differences between means (*p* < 0.05).

**Table 9:** Starch digestibility and predicted glycemic index.

Factors	0%	5% PPP	10% PPP	15% PPP
RDS (%)	62.29 ± 0.36 a	58.45 ± 0.20 b	53.29 ± 0.15 c	48.47 ± 0.26 d
SDS (%)	21.3 ± 1.0 d	23.8 ± 1.1 c	26.4 ± 1.2 b	28.9 ± 1.3 a
RS (%)	6.2 ± 0.4	7.5 ± 0.5	9.1 ± 0.6	10.8 ± 0.7
PGI	71.2 ± 1.8	67.4 ± 1.6	63.5 ± 1.5	58.7 ± 1.7

Abbreviations: PPP—pumpkin peel powder, RDS—rapidly digestible starch, SDS—slowly digestible starch, RS—resistant starch, pGI—predicted glycemic index. Values are expressed as mean ± standard deviation. Different superscript letters (a–d) within the same row indicate significant differences between means (*p* < 0.05).

**Table 10:** Bioaccessibility of phenolic compounds (in vitro digestion).

Factors	0%	5% PPP	10% PPP	15% PPP
Bioaccessible phenolics (%)	48.2 ± 2.1 d	52.6 ± 2.3 c	56.8 ± 2.4 b	60.3 ± 2.6 a
Antioxidant activity after digestion (µmol TE/g)	3.2 ± 0.2 d	6.4 ± 0.3 c	10.8 ± 0.5 b	14.6 ± 0.6 a

Abbreviations: PPP—pumpkin peel powder. Values are expressed as mean ± standard deviation. Different superscript letters (a–d) within the same row indicate significant differences between means (*p* < 0.05).

**Table 11:** Oxidative stability during storage of pasta enriched with PPP (30 days).

Factors	0%	5% PPP	10% PPP	15% PPP
PV (meq O_2_/kg fat)	3.8 ± 0.2 a	3.2 ± 0.2 b	2.7 ± 0.1 c	2.3 ± 0.1 d
TBARS (mg MDA/kg)	0.82 ± 0.04 a	0.71 ± 0.03 b	0.62 ± 0.03 c	0.55 ± 0.02 d

Abbreviations: PPP, pumpkin peel powder; PV, peroxide value; TBARS, thiobarbituric acid reactive substances; MDA, malondialdehyde. Values are expressed as mean ± standard deviation. Different superscript letters (a–d) within the same row indicate significant differences between means (*p* < 0.05).

**Table 12:** Effect of pumpkin peel powder (PPP) supplementation on the growth of probiotic bacteria.

Factors	0%	5% PPP	10% PPP	15% PPP
*Lactobacillus* growth (log CFU/mL)	7.2 ± 0.2 d	7.8 ± 0.2 c	8.3 ± 0.3 b	8.7 ± 0.3 a
*Bifidobacterium* growth (log CFU/mL)	7.0 ± 0.2 d	7.6 ± 0.2 c	8.1 ± 0.3 b	8.5 ± 0.3 a

Abbreviations: PPP, pumpkin peel powder. Values are expressed as mean ± standard deviation. Different superscript letters (a–d) within the same row indicate significant differences between means (*p* < 0.05).

## Data Availability

The data that support the findings of this study are available from the corresponding author, A.K.-D., upon reasonable request.

## Abbreviations

The following abbreviations are used in this manuscript:

PPP	pumpkin peel powder
DPPH	2,2-diphenyl-1-picrylhydrazyl
ABTS	2,2′-Azino-bis(3-ethylbenzothiazoline-6-sulfonic acid)
WHC	water-binding capacity
OHC	oil-holding capacity
G′	elastic modulus
G″	loss modulus
pGI	predicted glycemic index
RDS	rapidly digestible starch
SDS	slowly digestible starch
RS	resistant starch
TE	troxol equivalent
TPA	texture profile analysis
PV	peroxide value
TBARS	thiobarbituric acid reactive substances
MDA	malondialdehyde
CFU	colony-forming unit
PCA	principal component analysis
